# IL-12 signaling drives the differentiation and function of a T_H_1-derived T_FH1_-like cell population

**DOI:** 10.1038/s41598-019-50614-1

**Published:** 2019-09-30

**Authors:** Michael D. Powell, Kaitlin A. Read, Bharath K. Sreekumar, Devin M. Jones, Kenneth J. Oestreich

**Affiliations:** 1 Fralin Biomedical Research Institute at Virginia Tech Carilion, Roanoke, VA USA; 2 Department of Biomedical Sciences and Pathobiology, Virginia-Maryland Regional College of Veterinary Medicine, Virginia Tech, Blacksburg, VA USA; 3 Virginia Tech Carilion School of Medicine, Roanoke, VA USA; 4 Biomedical and Veterinary Sciences Graduate Program, Virginia Tech, Virginia, USA; 5 Translational Biology, Medicine, and Health Graduate Program, Virginia Tech, Virginia, USA

**Keywords:** Gene regulation in immune cells, Transcription

## Abstract

CD4^+^ T follicular helper (T_FH_) cells provide help to B cells and promote antibody-mediated immune responses. Increasing evidence supports the existence of T_FH_ populations that secrete cytokines typically associated with the effector functions of other CD4^+^ T cell subsets. These include T helper 1 (T_H_1)-biased T_FH_ (T_FH1_) cells that have recognized roles in both immune responses to pathogens and also the pathogenesis of autoimmune disease. Given their apparent importance to human health, there is interest in understanding the mechanisms that regulate T_FH1_ cell formation and function. However, their origin and the molecular requirements for their differentiation are unclear. Here, we describe a population of murine T_H_1-derived, T_FH1_-like cells that express the chemokine receptor Cxcr3 and produce both the T_H_1 cytokine interferon-γ and the T_FH_-associated cytokine interleukin-21 (IL-21). Furthermore, these T_FH1_-like cells promote B cell activation and antibody production at levels indistinguishable from conventional IL-6-derived T_FH_-like cells. Regarding their regulatory requirements, we find that IL-12 signaling is necessary for the differentiation and function of this T_FH1_-like cell population. Specifically, IL-12-dependent activation of STAT4, and unexpectedly STAT3, promotes increased expression of IL-21 and the T_FH_ lineage-defining transcription factor Bcl-6 in T_FH1_-like cells. Taken together, these findings provide insight into the potential origin and differentiation requirements of T_FH1_ cells.

## Introduction

Naïve T helper cells differentiate into a number of effector subsets that coordinate pathogen-specific immune responses including T helper 1 (T_H_1) and T follicular helper (T_FH_) cell populations. T_H_1 cells mediate immune responses to intracellular pathogens in large part through the production of the pro-inflammatory cytokine interferon-γ (IFN-γ), while T_FH_ cells aid in antibody-mediated immunity by activating B cells via cognate cell-cell interactions and secretion of the cytokine IL-21^1–3^. As with other T helper cell subsets, the differentiation of T_H_1 and T_FH_ cells is coordinated by the interplay between cell-extrinsic cytokine signals and cell-intrinsic transcription factor networks. For T_H_1 cells, IL-12-dependent activation of Signal Transducer and Activator of Transcription 4 (STAT4) drives the expression of the transcriptional regulator T-bet and expression of the T_H_1 gene program, while IL-6- or IL-21-dependent activation of STAT3 and up-regulation of the transcriptional repressor Bcl-6 play important roles in T_FH_ cell differentiation^4–11^.

Early work regarding T_H_1 and T_FH_ cells suggested that static populations of these cells developed in parallel during immune responses. However, it is now generally accepted that many T helper cell subsets are subject to phenotypic plasticity driven by signals from complex cytokine milieus during the course of an immune response^12–18^. With regard to T_FH_ populations, recent reports from both murine and human studies describe ‘hybrid’ T_FH_ cells that exhibit both B cell helper activity and secretion of effector cytokines normally expressed by other T helper subsets^19^. These include T_H_1-biased ‘T_FH1_’ cells, which are capable of producing the T_H_1 cytokine IFN-γ in addition to IL-21. These cells have been identified as important responders in murine infection models, and have also been described in humans infected with HIV, mycobacterium tuberculosis, and plasmodium, among other pathogens^20–27^. Additionally, cell populations phenotypically similar to T_FH1_ cells have been implicated in the onset of autoimmune disease, including systemic lupus erythematosus^28^. To date, however, the origin of these cells, including the regulatory mechanisms that direct both their differentiation and function, remains enigmatic.

Here, we describe the step-by-step *in vitro* differentiation of a murine T_H_1-derived, T_FH1_-like cell population that exhibits phenotypic and functional characteristics similar to T_FH1_ cells observed *in vivo*, in both murine and human settings. Specifically, these cells express elevated levels of Cxcr3 and are capable of producing both IFN-γ and IL-21. Interestingly, we find that T_FH1_-like cells provide B cell help similar to conventional T_FH_-like cells generated in the presence of IL-6. Mechanistically, we find that the differentiation and function of T_FH1_-like cells requires IL-12-dependent activation of both STAT4 and STAT3, which cooperatively drive the expression of both Bcl-6 and IL-21. Finally, and somewhat surprisingly, we found that while STAT3 activation required signals from IL-12, it was independent of autocrine IL-21 signaling. Taken together, the findings presented here provide insight into the potential origin and differentiation requirements of recently described T_FH1_ cell populations that have increasingly recognized roles in host immune responses and autoimmune disease.

## Results

### T_H_1-derived T_FH1_-like cells express both T-bet and Bcl-6

Increasing evidence suggests that T_FH_ cells exhibit considerable heterogeneity, and that phenotypically distinct T_FH_ subsets arise in response to diverse immune challenges. These include T_H_1-biased T_FH1_ cells, which secrete the cytokines IFN-γ and IL-21 and express the chemokine receptor Cxcr3^25,29^. While T_FH1_ cells have been observed in a number of clinical and experimental settings *in vivo*, the regulatory mechanisms underlying their development remain unclear^20,22,27,29^. A previous study from our laboratory demonstrated that T_H_1 cells upregulate a T_FH_-like gene program in response to decreased signals from environmental IL-2 (Supplementary Fig. 1A and^17^). These findings were in agreement with several other studies demonstrating that IL-2 signaling is a potent repressor of T_FH_ cell differentiation^30–32^. Given the T_H_1 origin of this T_FH_-like population, we sought to determine whether these cells were phenotypically and functionally similar to T_FH1_ cells described *in vivo*.

**Figure 1:**
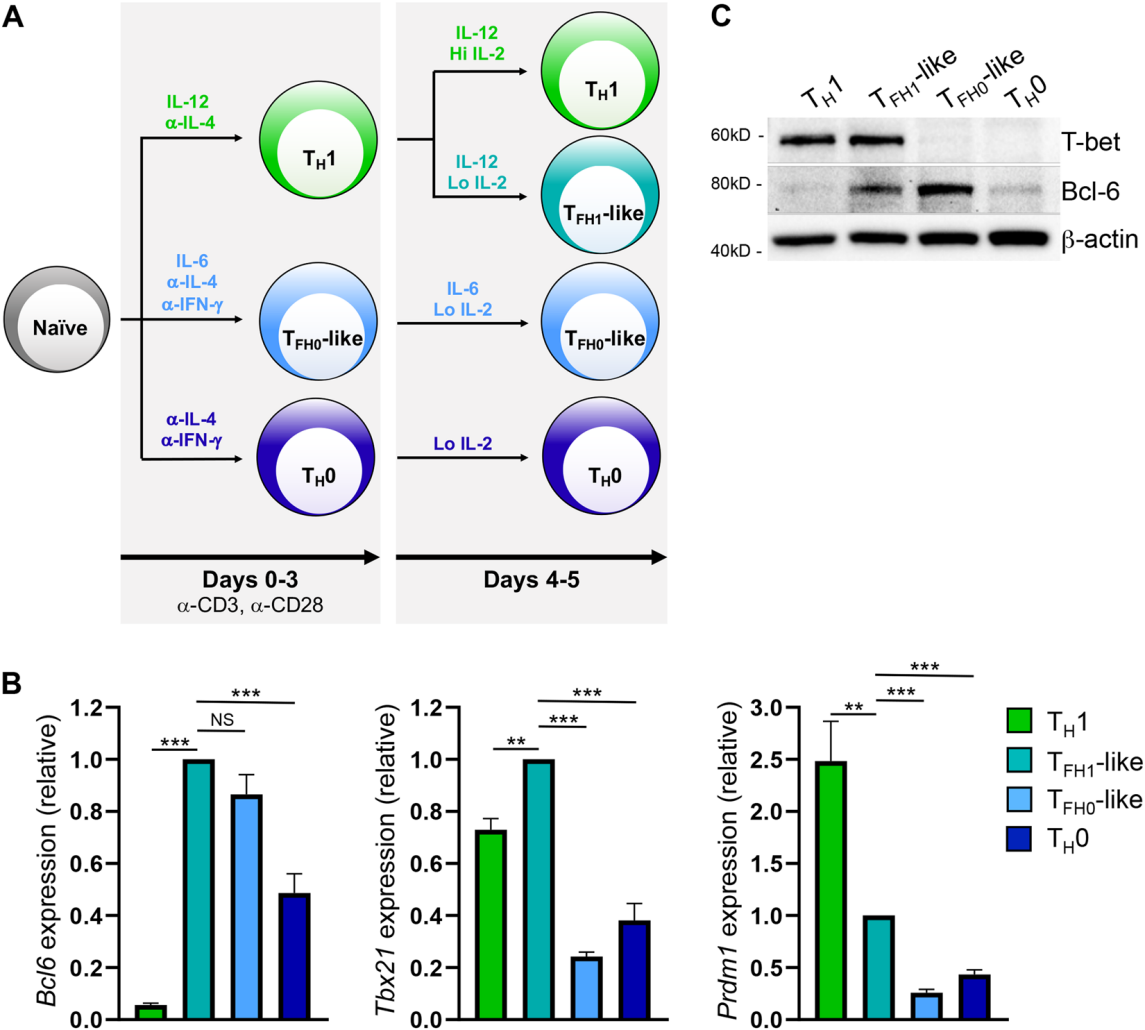
TFH1-like cells derived from TH1 cells uniquely co-express Bcl-6 and T-bet. (**A**) Schematic depicting culturing conditions utilized for the differentiation of the indicated cell populations. Briefly, naïve CD4^+^ T cells were cultured on plate-bound anti-CD3 and anti-CD28 as follows: T_H_1 (5 ng/mL IL-12, 5 μg/mL anti-IL-4), T_FH0_-like (50 ng/mL IL-6, 10 μg/mL anti-IL-4, 10 μg/mL anti-IFN-γ), or T_H_0 (10 μg/mL anti-IL-4, 10 μg/mL anti-IFN-γ). After 3 days, cells were removed from stimulation and plated under the following conditions: T_H_1 (5 ng/mL rmIL-12, 2.5 μg/mL anti-IL-4, 500 U/mL rhIL-2), T_FH1_-like (expanded from T_H_1 population; 5 ng/mL rmIL-12, 2.5 μg/mL anti-IL-4, 10 U/mL rhIL-2), T_FH0_-like (10 μg/mL anti-IL-4, 10 μg/mL anti-IFN-γ, 50 ng/mL rmIL-6, 10 U/mL rhIL-2), or T_H_0 (10 μg/mL anti-IL-4, 10 μg/mL anti-IFN-γ, 10 U/mL IL-2) for an additional 2 days. (**B**) qRT-PCR was used to assess expression of the indicated genes. The data were normalized to *Rps18* and presented as fold change relative to the T_FH1_-like sample (mean of *n* = 4–7 ± s.e.m.). **P < 0.01, ***P < 0.001; one-way ANOVA with Tukey multiple-comparison test. (**C**) Immunoblot analysis of Bcl-6 and T-bet protein expression in the indicated T helper cell populations. β-actin serves as a loading control. Shown is a representative blot of four independent experiments.

A hallmark feature of T_FH_ cell populations is their elevated expression of the transcriptional repressor Bcl-6^33–35^. As such, we began by comparing Bcl-6 expression in *in vitro*-differentiated murine T_H_1 cells, T_H_1-derived T_FH_-like (‘T_FH1_-like’) cells, previously described conventional T_FH_-like cells differentiated in the presence of IL-6 (‘T_FH0_-like’), and non-polarized T_H_0 cells^36^ (Fig. 1A). Relative to T_H_1 and T_H_0 cells, both T_FH1_-like and T_FH0_-like populations displayed elevated expression of the T_FH_ lineage-defining transcription factor Bcl-6 at both the transcript and protein level (Fig. 1B,C). In contrast, the Bcl-6 antagonist Blimp-1 was highly expressed only in the T_H_1 cell population (Fig. 1B). In addition to Bcl-6, T_FH1_ cells have also been shown to express the T_H_1 lineage-defining transcription factor T-bet^18,37^. As such, we next assessed T-bet expression levels across the above cell populations. Indeed, both transcript and protein analyses revealed that only the T_FH1_-like population expressed both T-bet and Bcl-6 (Fig. 1B,C). Collectively, these data demonstrate that T_H_1-derived T_FH1_-like cells express both Bcl-6 and T-bet similar to findings from T_FH1_ cells observed *in vivo*^18,37^.

### T_FH1_-like cells express elevated levels of Cxcr3, ICOS, and CD40 ligand

**Figure 2:**
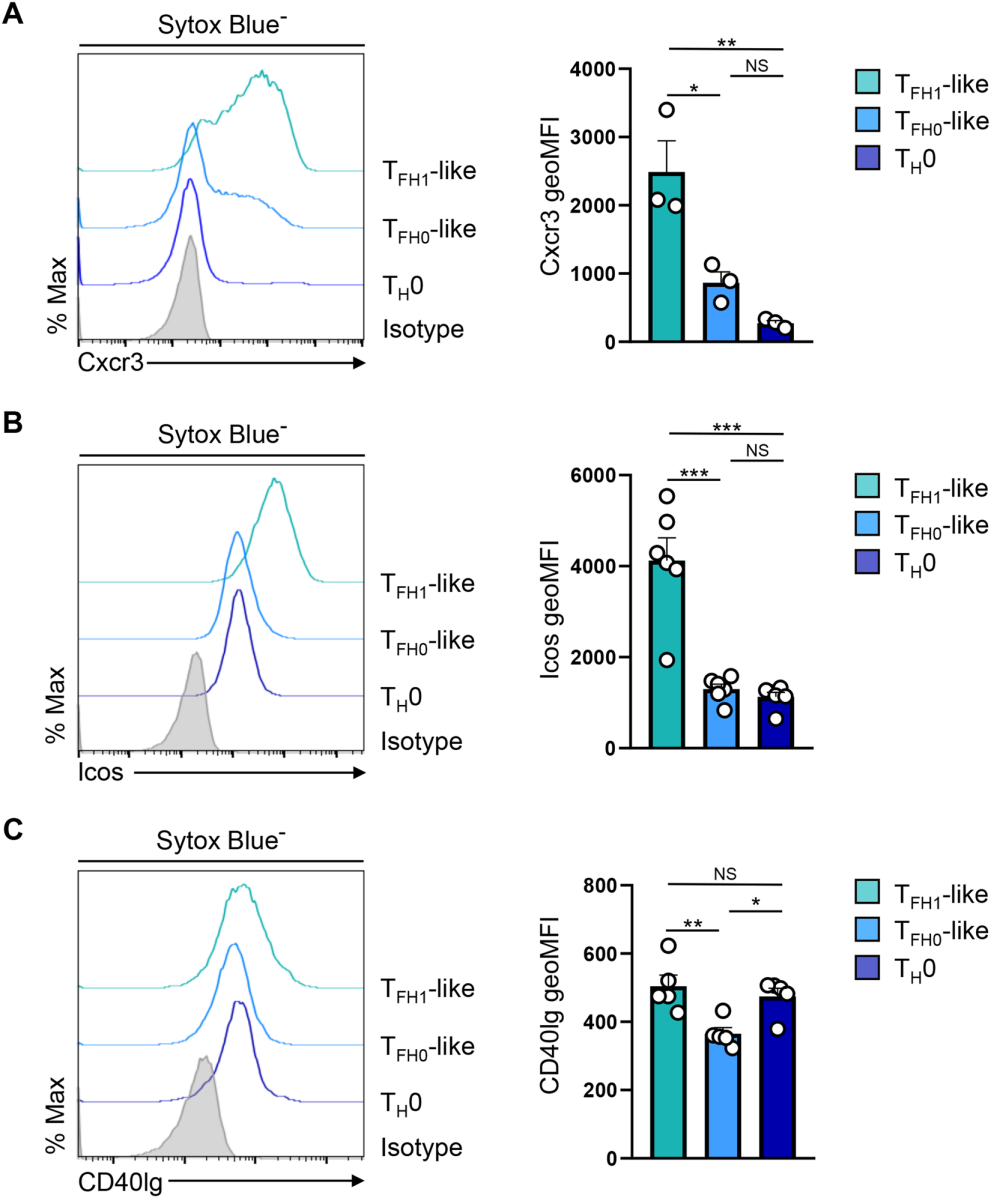
T_FH1_-like cells express elevated levels of Cxcr3, ICOS, and CD40 ligand. (**A**) Flow cytometry analysis of Cxcr3 surface expression on the indicated T helper cell populations. Mean fluorescence intensity (MFI) is also shown (mean of *n* = 3 ± s.e.m.). (**B**) Flow cytometry analysis of ICOS and CD40 ligand surface expression by the indicated T helper cell populations. Mean fluorescence intensity (MFI) is also shown (mean of *n* = 5–6 ± s.e.m.). *P < 0.05, **P < 0.01, ***P < 0.001; one-way ANOVA with Tukey multiple-comparison test.

A second distinguishing feature of T_FH1_ cells found *in vivo* is elevated surface expression of the chemokine receptor Cxcr3^24,25^. Indeed, gene expression analysis demonstrated that T_FH1_-like cells had elevated levels of *Cxcr3* expression compared to their T_H_1 cell counterparts (Supplementary Fig. 1B). Therefore, we next used flow cytometric analysis to determine the relative expression of Cxcr3 on the surface of T_FH1_-like and T_FH0_-like populations. Indeed, T_FH1_-like cells exhibited significantly more surface expression of Cxcr3 compared to the T_FH0_ population (Fig. 2A). Interestingly, two cell surface proteins that are critical for the B cell helper activity of T_FH_ cells, ICOS and CD40 ligand, were also more highly expressed on T_FH1_-like cells (Fig. 2B,C). To determine whether there were further differences in expression of the T_FH_ gene program between the T_FH1_- and T_FH0_-like populations, we performed additional transcript analyses and found that, while there was no significant difference in the expression of *Bcl6* or *Btla*, a number of other T_FH_-associated genes, including the chemokine receptor *Cxcr5*, were more highly expressed in the T_FH0_-like population (Fig. 1A and Supplementary Fig. 2A,B). Conversely, as with ICOS and CD40 ligand, T_FH1_-like cells expressed significantly higher levels of T_FH_ genes known to be critical for effective B cell helper activity (Supplementary Fig. 2C). Taken together, these findings demonstrate that T_FH1_-like cells preferentially express Cxcr3 alongside a number of proteins required to provide T cell help to B cells.

### T_FH1_-like cells produce both IFN-γ and IL-21

**Figure 3:**
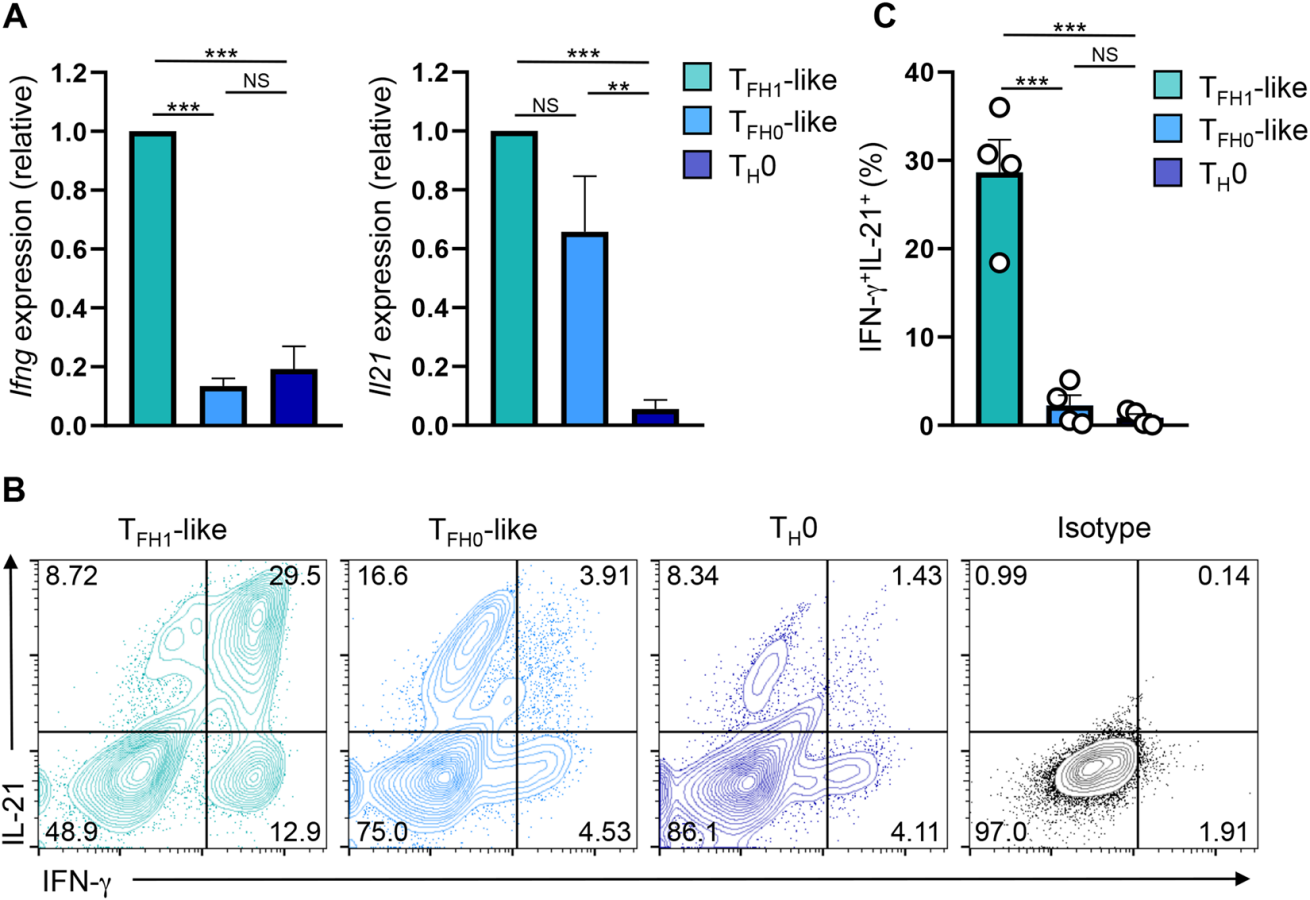
T_FH1_-like cells express both IFN-γ and IL-21. (**A**) qRT-PCR analysis of *Ifng* and *Il21* expression in the indicated cell populations following stimulation with PMA/Ionomycin for 2.5 hrs. The data were normalized to *Rps18* and presented as fold change relative to the T_FH1_-like sample (mean of *n* = 5 ± s.e.m.). (**B**) Flow cytometry analysis of intracellular expression of IL-21 and IFN-γ in the indicated cell populations. Shown are representative data from four independent experiments. (**C**) Percentage of IFN-γ^+^IL-21^+^ cells as assessed by flow cytometry analysis (mean of *n* = 4 ± s.e.m.). **P < 0.01, ***P < 0.001; one-way ANOVA with Tukey multiple-comparison test.

A functional characteristic of T_FH1_ cell populations is their expression of both IL-21 and IFN-γ^29,38^. To determine whether T_FH1_-like cells similarly exhibit this function, we evaluated their ability to simultaneously produce IL-21 and IFN-γ via flow cytometry. While both T_FH1_-like and T_FH0_-like populations expressed IL-21, T_FH1_-like cells were superior producers of IFN-γ (Fig. 3A,B). Importantly, a significantly higher percentage of T_FH1_-like cells were IFN-γ^+^IL-21^+^, as compared to the T_FH0_-like population (Fig. 3C). Interestingly, we did not observe significant differences in the expression of either IFN-γ or IL-21 between T_H_1 and T_FH1_-like cells (Supplementary Fig. 1C). This was interesting, as it has been reported that IFN-γ expression is subject to Bcl-6-dependent repression during the differentiation of conventional T_FH_ cell populations^35^. Collectively, these data demonstrate that in addition to exhibiting a T_FH1_ phenotype, T_H_1-derived T_FH1_-like cells also exhibit functional characteristics associated with T_FH1_ cells in the form of dual production of IFN-γ and IL-21^18,25^.

### T_FH1_-like cells are capable of activating B cells and inducing antibody production

**Figure 4:**
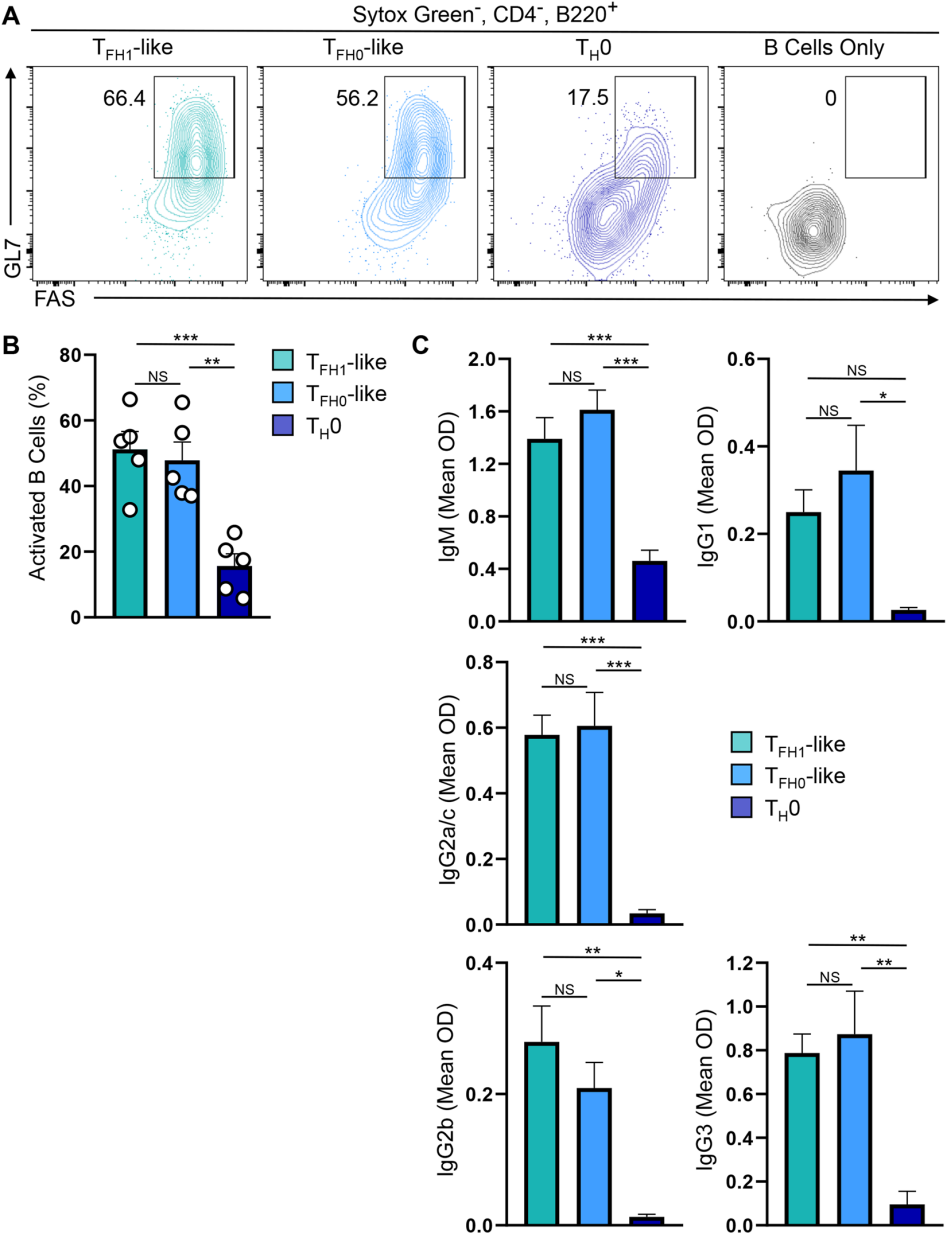
T_FH1_-like cells activate B cells and induce antibody production. (**A**) B cells were cultured with the indicated cell population (3:1 B/T ratio) and activation status was assessed by flow cytometry analysis of GL7 and FAS expression. Shown is representative data from five independent experiments. (**B**) The percentage of activated B cells (FAS^+^GL7^+^ cells) as assessed by flow cytometry in ‘A’ (mean of *n* = 5 ± s.e.m.). (**C**) ELISA analysis of antibody production by B cells cultured with the indicated cell population (3:1 B/T ratio) for 5 days (mean OD of *n* = 5 ± s.e.m.). **P < 0.01, ***P < 0.001; one-way ANOVA with Tukey multiple-comparison test.

To extend our functional analyses, we next compared B cell helper activity between the two T_FH_-like populations. Consistent with their increased IL-21 production and expression of T_FH_ cell markers, both T_FH_-like cell populations were more effective at promoting B cell activation than non-polarized T_H_0 cells (Fig. 4A,B). Furthermore, co-culture experiments demonstrated that both T_FH_-like populations were capable of inducing antibody production by B cells, while T_H_0 cells were poor providers of B cell help (Fig. 4C). Interestingly, we did not observe differences in the ability of T_FH0_-like and T_FH1_-like cells to preferentially induce isotype switching. This may be due to the presence of multiple cytokines in the culture media, including both IL-21 and IFN-γ, as well as others that were not analyzed (e.g. IL-10). Regardless, our data support a functional role for T_FH1_-like cells, similar to that of T_FH0_-like cells, in supporting B cell antibody production (Fig. 4C). Together, these data demonstrate that T_H_1-derived T_FH1_-like cells are capable of performing functions attributed to *bona fide* T_FH_ cells and, interestingly, are functionally similar to more conventional IL-6-derived T_FH_ cells^19,25,27^.

### T_FH_ gene expression patterns and B cell helper activity are dependent upon IL-12

**Figure 5:**
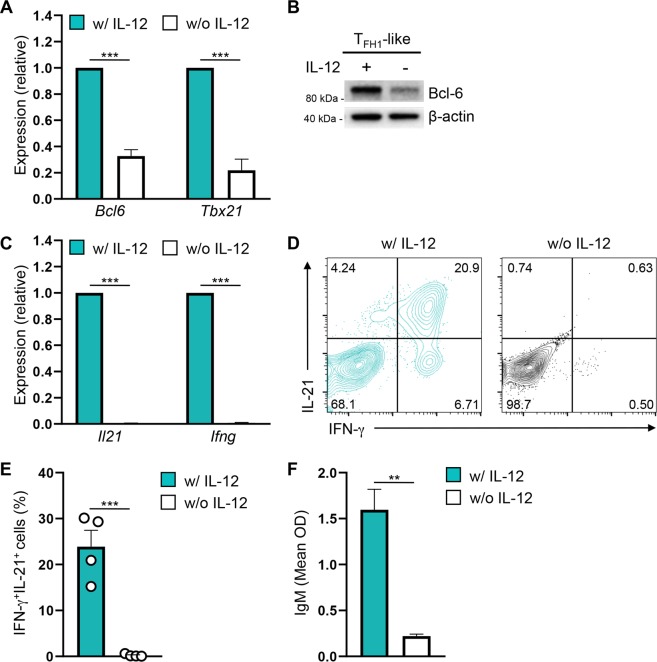
IL-12 signaling promotes Bcl-6, IL-21, and ICOS expression in T_FH1_-like cells. (**A**) qRT-PCR to assess expression of the indicated genes in T_FH1_-like cells cultured with (teal bars) or without (white bars) IL-12. The data were normalized to *Rps18* and presented as fold change relative to T_FH1_-like cells cultured with IL-12 (mean of *n* = 3 ± s.e.m.). (**B**) Immunoblot analysis of Bcl-6 protein expression in T_FH1_-like cells cultured with or without IL-12. Shown is a representative blot of three independent experiments. β-actin was used as a loading control. (**C**) qRT-PCR to assess expression of the indicated genes in T_FH1_-like cells cultured with (blue bars) or without (white bars) IL-12. The data were normalized to *Rps18* and presented as fold change relative to T_FH1_-like cells cultured with IL-12 (mean of *n* = 3 ± s.e.m.). (**D**) Flow cytometry analysis of intracellular expression of IL-21 and IFN-γ in T_FH1_-like cells cultured with or without IL-12. Shown is representative data from four independent experiments. (**E**) The percent of IFN-γ^+^IL-21^+^ cells as assessed by flow cytometry analysis in ‘D’ (mean of *n* = 4 ± s.e.m.). (**F**) ELISA analysis of IgM production by B cells co-cultured with the indicated T_FH1_-like population at a 3:1 B/T cell ratio for 5 days (mean OD of *n* = 3 ± s.e.m.). **P < 0.01, ***P < 0.001; unpaired Student’s *t*-test.

We next sought to determine specific cytokine signals and transcription factors responsible for driving the T_FH1_-like phenotype. A notable difference between the T_FH1_-like and T_FH0_-like cell populations is that the T_H_1-derived T_FH1_-like cells are cultured in the presence of IL-12, rather than IL-6. While IL-12 has been reported to be an important factor in the *in vitro* and *in vivo* differentiation of human T_FH_ cell populations, the role of IL-12 in promoting murine T_FH_ cell differentiation is less clear^39–42^. In order to assess the role of IL-12 in T_FH1_-like cell differentiation, we cultured T_FH1_-like cells with and without IL-12 and assessed their expression of notable T_FH1_ cell transcription factors and cell surface receptors. Strikingly, expression of both T-bet and Bcl-6 was significantly reduced in the absence of IL-12 (Fig. 5A,B). Additional analyses revealed that while many T_FH_ genes were unaffected by the loss of IL-12, the expression of the key T_FH_-associated gene *Icos* was significantly reduced at both the transcript and protein level (Supplementary Fig. 3).

We next wanted to assess the importance of IL-12 signaling to T_FH1_-like cell function. As such, we assessed the ability of T_FH1_-like cells cultured in the presence and absence of IL-12 to produce cytokines and induce B cell-mediated antibody production. Importantly, production of IL-21 and IFN-γ, as well as the percentage of IFN-γ^+^IL-21^+^ cells, was reduced in the absence of signals from IL-12 (Fig. 5C–E). Furthermore, T_FH1_-like cells cultured without IL-12 were poor inducers of B cell antibody production as compared to IL-12-cultured controls (Fig. 5F). Taken together, these data demonstrate that IL-12 signaling is a potent inducer of the phenotypic and functional properties of T_FH1_-like cells.

### STAT4 and STAT3 associate with the *Bcl6* and *Il21* loci downstream of IL-12 signaling

**Figure 6:**
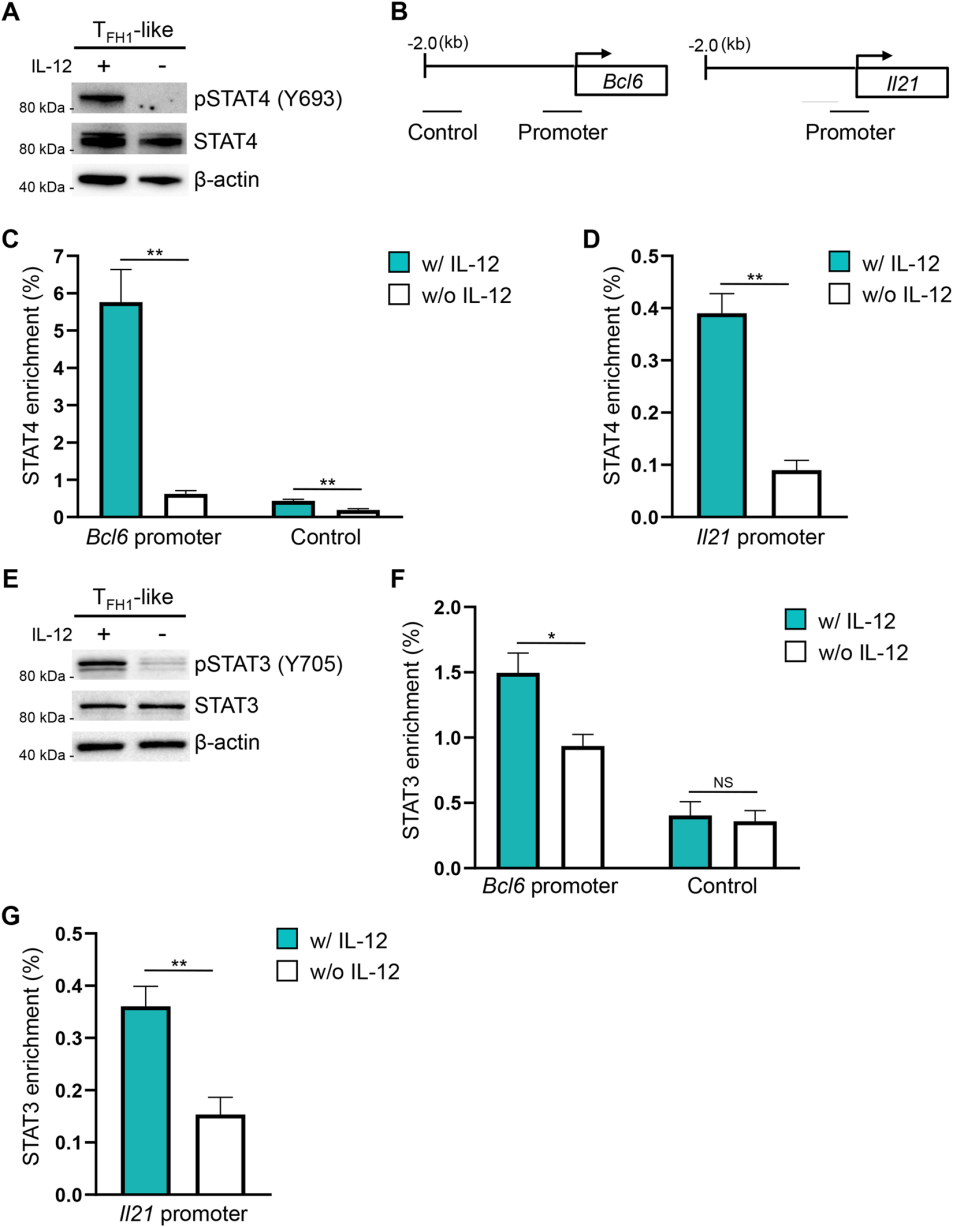
IL-12 signaling results in increased association of STAT4 and STAT3 with the *Bcl6* and *Il21* loci. (**A**) Immunoblot analysis to assess STAT4 activation (pSTAT4 Y693) in T_FH1_-like cells cultured with or without IL-12. STAT4 and β-actin serve as controls for total STAT4 and equal protein loading, respectively. Shown is a representative blot of three independent experiments. (**B**) Schematic depicting the location of amplicons within the *Bcl6* and *Il21* gene loci utilized for ChIP analyses. (**C**,**D**) ChIP assays to evaluate STAT4 enrichment at the *Bcl6* and *Il21* loci in T_FH1_-like cells cultured with or without IL-12. Data are presented as percent enrichment relative to a “total” input sample (mean of *n* = 4 ± s.e.m.). (**E**) Immunoblot analysis of activated STAT3 (pSTAT3 Y705) in T_FH1_-like cells cultured with or without IL-12. STAT3 and β-actin are shown as controls for total STAT3 and equal protein loading, respectively. Shown is a representative blot from three independent experiments. (**F**,**G**) ChIP assays to quantify STAT3 enrichment at the *Bcl6* and *Il21* loci in T_FH1_-like cells cultured with or without IL-12. Data are presented as percent enrichment relative to a “total” input sample (mean of *n* = 4 ± s.e.m.). *P < 0.05, **P < 0.01; unpaired Student’s *t*-test.

We next sought to identify transcription factors downstream of IL-12 signaling that may regulate expression of key T_FH_ genes in T_FH1_-like cells. We began by focusing on STAT4, which is activated (phosphorylated) downstream of signals from IL-12 (Fig. 6A). A previous study described STAT4 association with the *Bcl6* and *Il21* loci^42^. Indeed, we observed increased STAT4 enrichment at both the *Bcl6* and *Il21* promoters in T_FH1_-like cells cultured in the presence of IL-12, as compared to cells cultured without IL-12 (Fig. 6B–D). These findings suggest that STAT4 is a positive regulator of both Bcl-6 and IL-21 expression in T_FH1_-like cells.

In addition to STAT4, STAT3 has also been identified as an important positive regulator of T_FH_ gene expression^5,17,43,44^. STAT3 is activated in response to signaling from a number of cytokines including IL-21. Our previous findings demonstrated that IL-12 signaling was a potent driver of IL-21 expression by the T_FH1_-like population. As such, we considered the possibility that STAT3 activation may also require upstream signals from IL-12 in T_FH1_-like cells. Indeed, we found that STAT3 activation was reduced in the absence of IL-12 (Fig. 6E). Consistent with these data, we observed decreased STAT3 enrichment at the *Bcl6* and *Il21* promoters in T_FH1_-like cells cultured without IL-12 (Fig. 6F,G). We next sought to determine whether the observed STAT3 activation was due to IL-12-dependent, autocrine IL-21 signaling. However, we observed no difference in STAT3 activation or in the expression of Bcl-6 when IL-21R-deficient cells were differentiated under T_FH1_-like polarizing conditions, suggesting that STAT3 activation downstream of IL-12 signals was independent of IL-21 (Supplementary Figs. 4A–C). Collectively, these data implicate cooperative, IL-12-dependent activities of both STAT4 and STAT3 in the differentiation and functional regulation of T_FH1_-like cells.

## Discussion

Recent work has established that T_H_1-biased T_FH_ cell populations exist *in vivo* and that these cells play roles in both healthy immune responses and autoimmune disease^20,24,25,28,29^. To date, the cytokine signals and transcriptional mechanisms underlying their formation and function are unclear. Here, we demonstrate that *in vitro*-generated T_H_1 cells are capable of differentiating into a T_FH1_-like cell population that exhibits phenotypic and functional characteristics associated with T_FH1_ cells. Similar to T_FH1_ cells described *in vivo*, T_FH1_-like cells express both the T_FH_ lineage-defining factor Bcl-6 and the T_H_1 lineage-defining factor T-bet, in addition to the chemokine receptor Cxcr3. Functionally, our findings demonstrate that the T_FH1_-like cell population is capable of producing both IFN-γ and IL-21 and providing help to B cells that results in B cell activation and antibody production.

While there is a general consensus on the phenotypic and functional properties of T_FH1_ cells, there is still a debate as to their cellular origin. The findings presented here support a developmental pathway wherein T_H_1 cells give rise to a hybrid T_FH1_-like cell state by inducing the expression of Bcl-6 and other aspects of the T_FH_ gene program. In agreement with our findings, a recent report demonstrated that IFN-γ-producing T_FH_ cells generated *in vivo* require prior expression of T-bet^37^. Alternatively, it has been suggested that T_FH1_ cells are conventional T_FH_ cells that gain T-bet expression and the ability to produce IFN-γ^29^. While our work does not necessarily support this model, it is important to note that our findings also do not exclude this as a potential mechanism. Future studies will be necessary to determine whether a dominant differentiation pathway for T_FH1_ cells exists or whether there may be multiple origins from which these specialized T_FH_ cell populations arise.

Our current findings also provide mechanistic insight into the potential cytokine signals and downstream transcriptional mechanisms required for the differentiation of T_FH1_ cells. In this regard, we find that the expression of a number of genes that promote induction of the T_FH1_-like cell state is dependent upon signals from the cytokine IL-12. Specifically, we find that IL-12 is required for the elevated expression of Bcl-6 and ICOS that is observed in the T_FH1_-like population, as well as the production of IFN-γ and IL-21. These data are in agreement with previous work in human cells demonstrating that IL-12 is required for the development of a CD4^+^ T cell population that co-expresses IFN-γ and IL-21^26^. Furthermore, our findings are also in agreement with work identifying a role for IL-12 in positively regulating the development and function of human T_FH_ cells in both *in vitro* and *in vivo* settings^40,41,43,45^.

Interestingly, while the role of IL-12 in human T_FH_ cell development is relatively well defined, the requirement for IL-12 signals in murine T_FH_ differentiation is less clear^16,18,42^. As a potential explanation for this discrepancy, we find that while IL-12 signaling in the form of STAT4 and STAT3 activation is required to drive the expression of a subset of T_FH_ genes, including *Bcl6*, *Icos*, and *Il21*, signals from IL-12 appear to play a complementary but secondary role to those derived from IL-2^17^. Thus, our findings support a model wherein combined IL-12 and IL-2 signals drive T_H_1 cell differentiation, while IL-12 signals in the absence of strong IL-2 signaling promote the alternative T_FH1_-like phenotype. Indeed, our previous work, and that of others, has demonstrated that the IL-2/STAT5 signaling axis functions as a potent negative regulator of T_FH_ cell development^17,30–32^.

Intriguingly, our work implicates an IL-12/STAT4/STAT3 signaling axis in the positive regulation of T_FH1_ cell development, as IL-12 signaling is required for both STAT4 and STAT3 activation in the T_FH1_-like population. In agreement with these findings, STAT4 activation has been previously implicated in the generation of IFNγ-producing T-bet^+^Bcl-6^+^ T_FH_ cells generated in response to viral infection^18^. Though our findings suggest that IL-12-dependent activation of STAT3 promotes the expression of the T_FH1_ phenotype, the identity of the signals that drive the phosphorylation of STAT3 remain unclear. Classically, IL-12 signaling has been associated with STAT4 activation. However, previous work has indicated that IL-12 signals are also capable of inducing STAT3 activation^46^. We also considered the possibility that STAT3 activation may arise from autocrine IL-21 signaling. However, experiments with IL-21R^−/−^ T cells demonstrated that STAT3 activation is independent of IL-21. Thus, whether STAT3 activation is occurring directly downstream of IL-12 signaling, or downstream of signals from an IL-12-dependent cytokine, is as yet unclear.

Collectively, the work presented here supports a possible T_H_1 origin for the T_FH1_ cells observed *in vivo*, and also provides insights into the regulatory requirements that govern their development. Given their role in infection and autoimmune disease, a better understanding of such regulatory requirements may identify potential therapeutic targets, which will allow for more selective manipulation of T_FH1_ cell populations in efforts to treat human disease.

## Methods

### Primary cells and cell culture

All mouse strains [C57BL/6J, C57BL/6NJ, IL-21R^−/−^ (C57BL/6NJ background)] were obtained from the Jackson Laboratory. Naïve CD4^+^ T cells were purified from the spleens and lymph nodes of 5–8 week old mice via negative selection using the BioLegend Mojosort kit according to the manufacturer’s instructions. For all experiments, cells were cultured in complete IMDM (“cIMDM”: IMDM [Life Technologies], 10% FBS [Life Technologies], 1% Penicillin-Streptomycin [Life Technologies], and 50 μM β-mercaptoethanol [Sigma-Aldrich]). Following isolation, cells were plated at a density of 3–5 × 10^5^ cells per well and stimulated using plate-bound anti-CD3ε (5 µg/ml; BD Biosciences) and anti-CD28 (10 μg/ml; BD Biosciences) under the following polarizing conditions: T_H_1 (5 ng/mL rmIL-12 [R&D Systems], 5 μg/mL anti-IL-4), T_FH0_-like (10 μg/mL anti-IFN-γ, 10 μg/mL anti-IL-4, 50 ng/mL rmIL-6 [R&D Systems]), T_H_0 (10 μg/mL anti-IFN-γ [XMG1.2; BioLegend], 10 μg/mL anti-IL-4 [11B11; BioLegend]). After 3 days, cells were removed from stimulation and expanded to plate at 5–7 × 10^5^ cells/well in fresh media under the following conditions: T_H_1 (5 ng/mL rmIL-12, 2.5 μg/mL anti-IL-4, 500 U/mL rhIL-2 [NIH]), T_FH1_-like (expanded from T_H_1 population; 5 ng/mL rmIL-12, 2.5 μg/mL anti-IL-4, 10 U/mL rhIL-2), T_FH0_-like (10 μg/mL anti-IFN-γ, 10 μg/mL anti-IL-4, 50 ng/mL rmIL-6, 10 U/mL rhIL-2), T_H_0 (10 μg/mL anti-IFN-γ, 10 μg/mL anti-IL-4, 10 U/mL IL-2) for an additional 48 h. Where indicated, IL-12 was omitted from the T_FH1_-like culturing conditions. The Institutional Animal Care and Use Committee of Virginia Tech approved all experiments involving the use of mice. All methods were performed in accordance with the approved guidelines.

### T and B cell co-culture and analysis of helper activity

B cells were purified from the spleens and lymph nodes of age- and sex-matched 5–8 week old C57BL/6J mice using the MojoSort Mouse Pan B cell isolation kit (BioLegend), according to the manufacturer’s instructions. For each indicated population, 1 × 10^5^ T cells were mixed with B cells at a 1:3 T cell:B cell ratio and stimulated using plate-bound anti-CD3ε (5 μg/mL) under T_FH1_-like or T_FH0_-like conditions. Where indicated, IL-12 was omitted from the T_FH1_-like conditions. T and B cells were co-cultured for either 2 days, at which point B cell activation was analyzed by flow cytometry, or 4 days, where supernatant was collected for ELISA analysis of antibody production.

Antibody production was measured using the BD Pharmingen Mouse Immunoglobulin Isotyping ELISA kit according to the manufacturer’s instructions. OD450 values were calculated by subtracting OD450 readings taken from supernatants from B cells cultured alone in the indicated polarizing conditions from the OD450 values of co-cultured samples.

### RNA isolation and qRT-PCR

RNA was purified using the NucleoSpin RNA Kit (Macherey-Nagel). Complementary DNA was generated using the Superscript IV First Strand Synthesis System (Thermo Fisher). qRT-PCR reactions were performed with 10–20 ng cDNA per reaction, gene-specific primers (*Rps18* forward: 5′-GGAGAACTCACGGAGGATGAG-3′, *Rps18* reverse: 5′-CGCAGCTTGTTGTCTAGACCG-3′; *Bcl6* forward: 5′-CCAACCTGAAGACCCACACTC-3′, *Bcl6* reverse: 5′-GCGCAGATGGCTCTTCAGAGTC-3′; *Tbx21* forward: 5′-GTGACTGCCTACCAGAACGC-3′, *Tbx21* reverse: 5′-AGGGGACACTCGTATCAACAG-3′; *Il21* forward: 5′-TGGATCCTGAACTTCTATCAGCTCC-3′, *Prdm1* forward: 5′-CTTGTGTGGTATTGTCGGGAC-3′, *Prdm1* reverse: 5′-CACGCTGTACTCTCTCTTGG-3′; *Il21* reverse: 5′-AGGCAGCCTCCTCCTGAGC-3′; *Ifng* forward: 5′-CTACCTTCTTCAGCAACAGC-3′, *Ifng* reverse: 5′-GCTCATTGAATGCTTGGCGC-3′; *Il21* forward: 5′- TGGATCCTGAACTTCTATCAGCTCC, *Il21* reverse: 5′- AGGCAGCCTCCTCCTGAGC; *Cxcr5* forward: 5′-GTACCTAGCCATCGTCCATGC-3′, *Cxcr5* reverse: 5′-GTGCACTGTGGTAAGGAGTCG-3′; *Btla* forward: 5′-CATCCCAGATGCCACCAATGC-3′, *Btla* reverse: 5′-CAGAAAGCAGAGCAGGCAGAC-3′; *Icos* forward: 5′-CTCACCAAGACCAAGGGAAGC-3′, *Icos* reverse: 5′-CCACAACGAAAGCTGCACACC-3′; *Cd40l* forward: 5′-AGCCAACAGTAATGCAGCATCCG-3′, *Cd40l* reverse: 5′-AGCCAGAGGCCGACGATGAATG-3′; *Sh2d1a* forward: 5′-CTGGATGGAAGCTATCTGCTG-3′, *Sh2d1a* reverse: 5′-CAGGTGCTGTCTCGGCACTCC-3′; *Il6ra* forward: 5′-CCACATAGTGTCACTGTGCG-3′, *Il6ra* reverse: 5′-GGTATCGAAGCTGGAACTGC-3′; *Tnfsf8* forward: 5′-GCAGCTACTTCTACCTCAGCAC-3′, *Tnfsf8* reverse: 5′-GTGCCATCTTCGTTCCATGACAG-3′; *Pdcd1* forward: 5′-CGTCCCTCAGTCAAGAGGAG-3′, *Pdcd1* reverse: 5′-GTCCCTAGAAGTGCCCAACA-3′; *Cxcr3* forward: 5′- CCTTGAGGTTAGTGAACGTC-3′, *Cxcr3* reverse: 5′-GCTGGCAGGAAGGTTCTGTC-3′) and SYBR Select Mastermix (Life Technologies). All samples were normalized to *Rps18* as a control and are presented either as mRNA relative to *Rps18*, or as fold change relative to the control sample, as indicated. To measure *Il21* and *Ifng* transcript expression, the indicated T helper populations were re-stimulated with PMA and Ionomycin for 2.5–4 h.

### Immunoblot analyses

Immunoblot analyses were performed as described previously^44^. Antibodies used to detect chosen proteins were as follows: Bcl-6 (1:500, BD Pharmingen), T-bet (1:1,000, Santa Cruz), pSTAT3 Y705 (1:20,000, Abcam), STAT3 (1:5000, Santa Cruz), pSTAT4 Y693 (1:1000, Cell Signaling), and STAT4 (1:2500, BioLegend). For all experiments, β-actin (1:15,000, GenScript) expression was monitored to ensure equivalent protein loading. Original and uncropped images of immunoblots can be found in Supplementary Fig. 5.

### Flow cytometry

For extracelluar epitopes, cells were harvested and washed 1x with FACS buffer (PBS w/2% FBS, 1% BSA) prior to staining with flurochrome-conjugated anti-ICOS PE (eBioscience), anti-CD40lg PE (Biolegend), anti-Cxcr3 PE (Biolegend), anti-FAS BV421 (BD Bioscience), anti-GL7 AF488 (BD Bioscience), anti-CD4 AF488 (R&D), anti-B220 PECy7 (BD Bioscience), anti-IL21R PE (Biolegend), or the appropriate isotype control. Cells were incubated for 1 hour at room temperature and subsequently washed 2x with FACS buffer. Cell viability was determined by incubating the cells with either Sytox Blue (ThermoFisher) or Sytox Green (ThermoFisher). Samples containing B cells were subjected to Fc block anti-CD16/CD32 (Invitrogen). For cytokine staining, cells were treated with Golgi stop (BD Bioscience) and restimulated with PMA and Ionomycin for 2 h. Cells were fixed and permeablized using the BD Cytofix/Cytoperm kit as detailed by the manufacturer. Cells were incubated with IL-21R subunit/Fc chimera (R&D Systems), washed with Perm/Wash buffer (BD), stained with F(ab’)2 anti-Human IgG Fc R-PE (Life Technologies), washed, and finally stained with anti-IFN-γ AF700 (R&D Systems). All incubations were carried out for 30 minutes at 4 °C. Goat F(ab’)2 IgG R-PE (Life Technologies) and Rat IgG2a AF700 (R&D Systems) were used as isotype controls. Samples were analyzed on the BD Accuri C6 or the Sony SH800 flow cytometers and data evaluated using Flowjo software.

### ChIP

Chromatin was prepared from the indicated T helper cell population as previously described^17^. The resulting chromatin was incubated with either anti-STAT4 (Santa Cruz), anti-STAT3 (Santa Cruz), or control antibody (Abcam) and immunoprecipitated using Protein G Dynabeads (Life Technologies). Precipitated DNA was analyzed via quantitative PCR using SYBR Select Mastermix (Life Technologies) and gene-specific primers: (*Bcl6* promoter forward: 5′-GCGGAGCAATGGTAA AGCCC-3′, and reverse: 5′-CTGGTGTCCGGCCTTTCCTAG-3′; *Bcl6* control forward: 5′-GTACTCCAACAACAGCACAGC-3′, and reverse: 5′-GTGGCTCGTTAAATCACAGAGG-3′; *Il21* promoter forward: 5′-CAC ACACCTTGGTGAATGCTG-3′, and reverse: 5′-CCATTGGCTAGGTGTACGTGTG-3′). Samples were normalized to total input followed by the subtraction of the isotype control to account for unspecific binding.

### Statistical analyses

All data represent at least three independent experiments. Error bars represent the standard error of the mean. For statistical analysis, unpaired *t* tests or one-way ANOVA with Tukey multiple comparison tests were performed to assess statistical significance, as appropriate for a given experiment. *P* values < 0.05 were considered statistically significant.

## Supplementary information

Supplementary Material

## Data Availability

The datasets produced in this study will be made available upon reasonable request. Requests should be sent to the corresponding author.
