# Supplementary material for: IL-12 signaling drives the differentiation and function of a TH1-derived TFH1-like cell population
Source: Sci Rep. 2019 Sep 30;9:13991. doi: 10.1038/s41598-019-50614-1 (PMC6769002; doi:10.1038/s41598-019-50614-1)
Supplement: Supplementary file 1 — Supplementary Material [file 41598_2019_50614_MOESM1_ESM.pdf]

Supplementary material associated with:

Title: IL-12 signaling drives the differentiation and function of a T_H1-derived T_{FH1}-like cell population

Authors: Michael D. Powell, Kaitlin A. Read, Bharath K. Sreekumar, Devin M. Jones & Kenneth J. Oestreich

Supplementary Figure 1

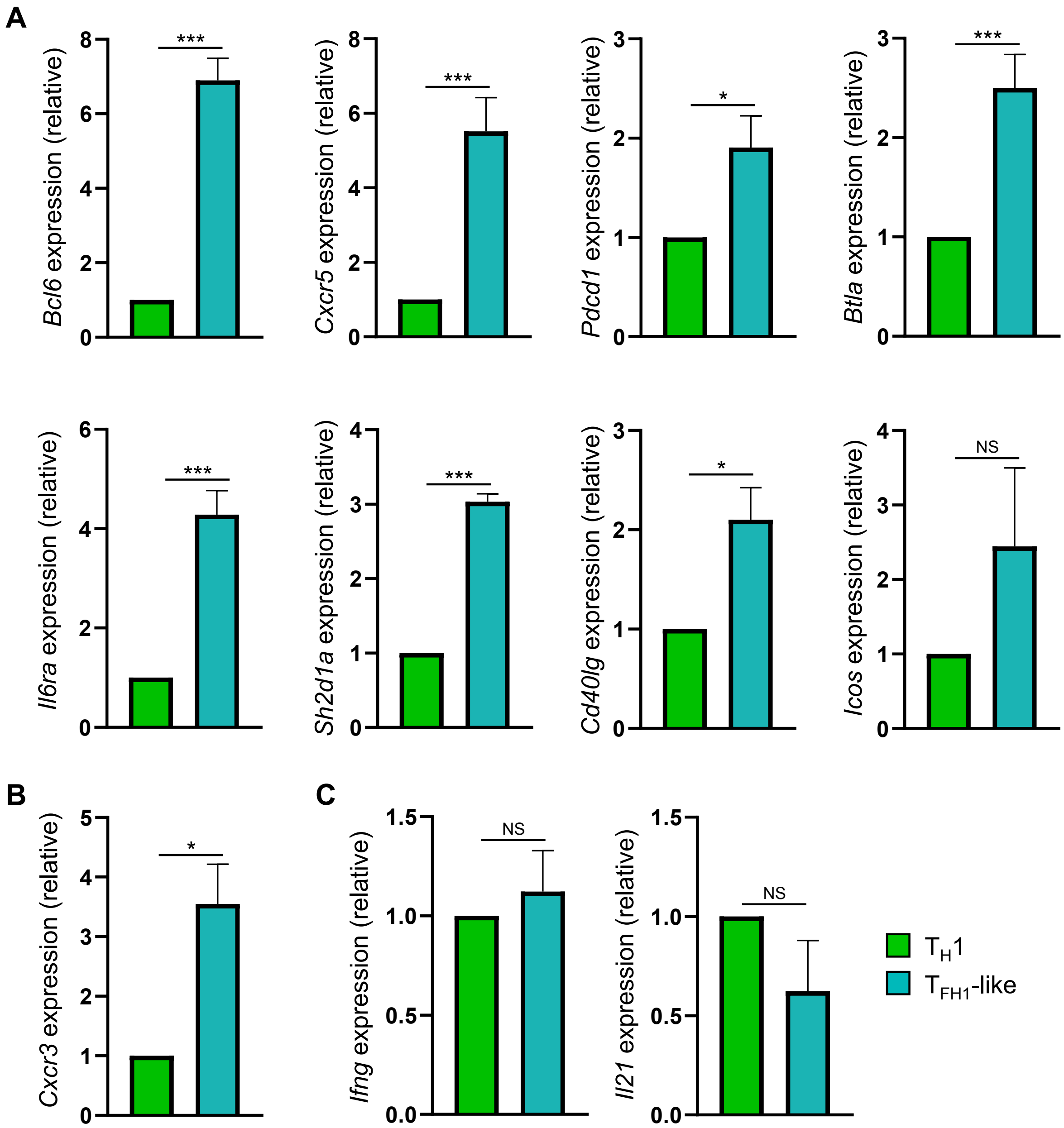

Supplementary Figure 1. T_H1-derived T_{FH1}-like cells express genes associated with T_{FH} differentiation and function. (A-C) qRT-PCR analysis of the indicated genes in T_H1 and T_{FH1}-like cells. For 'C', cells were stimulated for 2h with PMA and Ionomycin prior to harvest and analysis of gene expression. Samples were normalized to *Rps18* and are represented relative to the T_H1 sample (mean of n = 4-8 ± s.e.m.). *$P < 0.05$, **$P < 0.01$, ***$P < 0.001$; unpaired Student's *t*-test).

Supplementary Figure 2

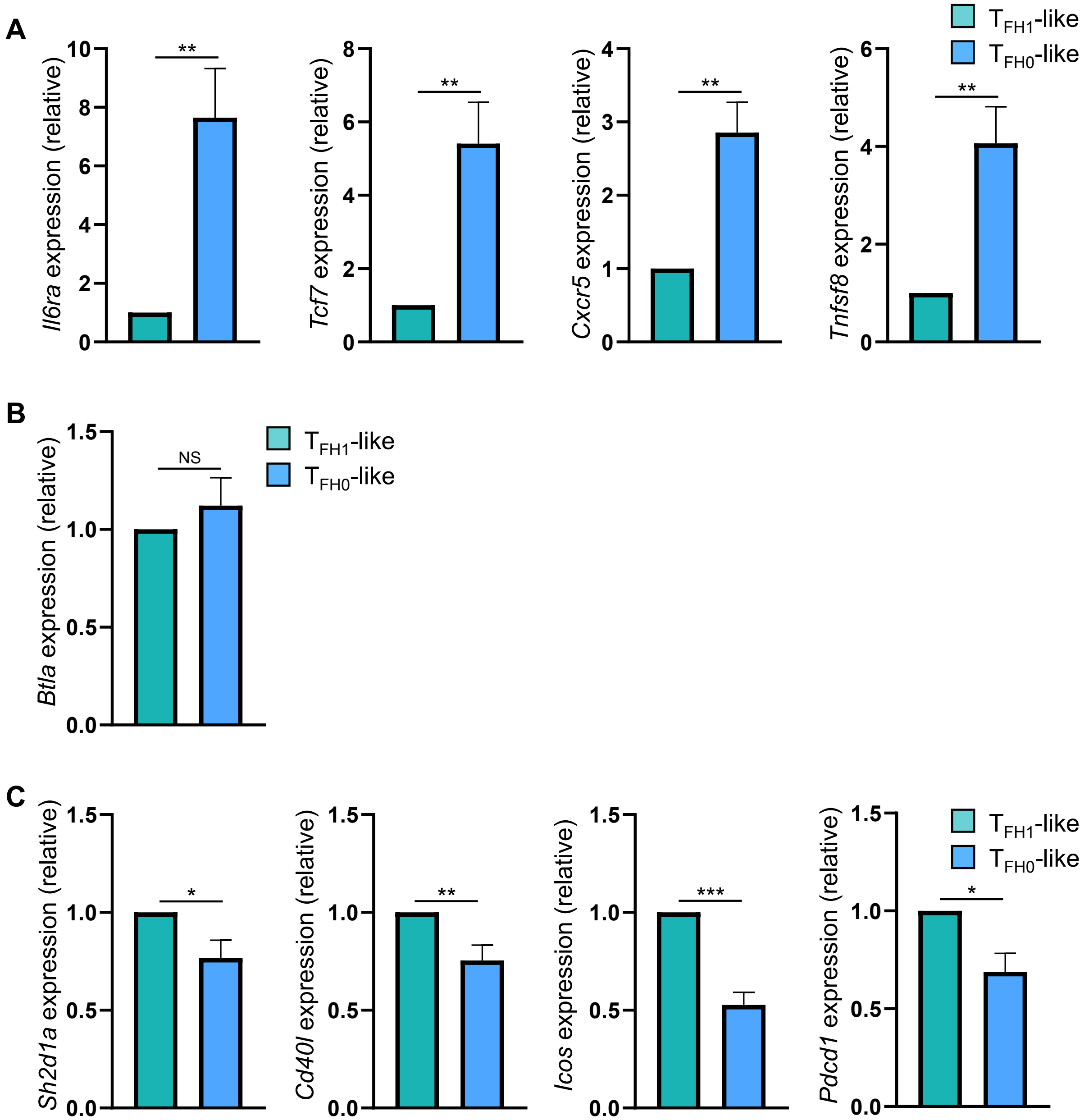

Supplementary Figure 2. T_{FH}-associated genes are differentially expressed between T_{FH1}- and T_{FH0}-like cell populations. (A-C) qRT-PCR analysis of the indicated genes in T_{FH1}-like and T_{FH0}-like cells. Samples were normalized to *Rps18* and are represented relative to the T_{FH1}-like sample (mean of n = 4-8 ± s.e.m.). *P < 0.05, **P < 0.01, ***P < 0.001; unpaired Student's t-test).

Supplementary Figure 3

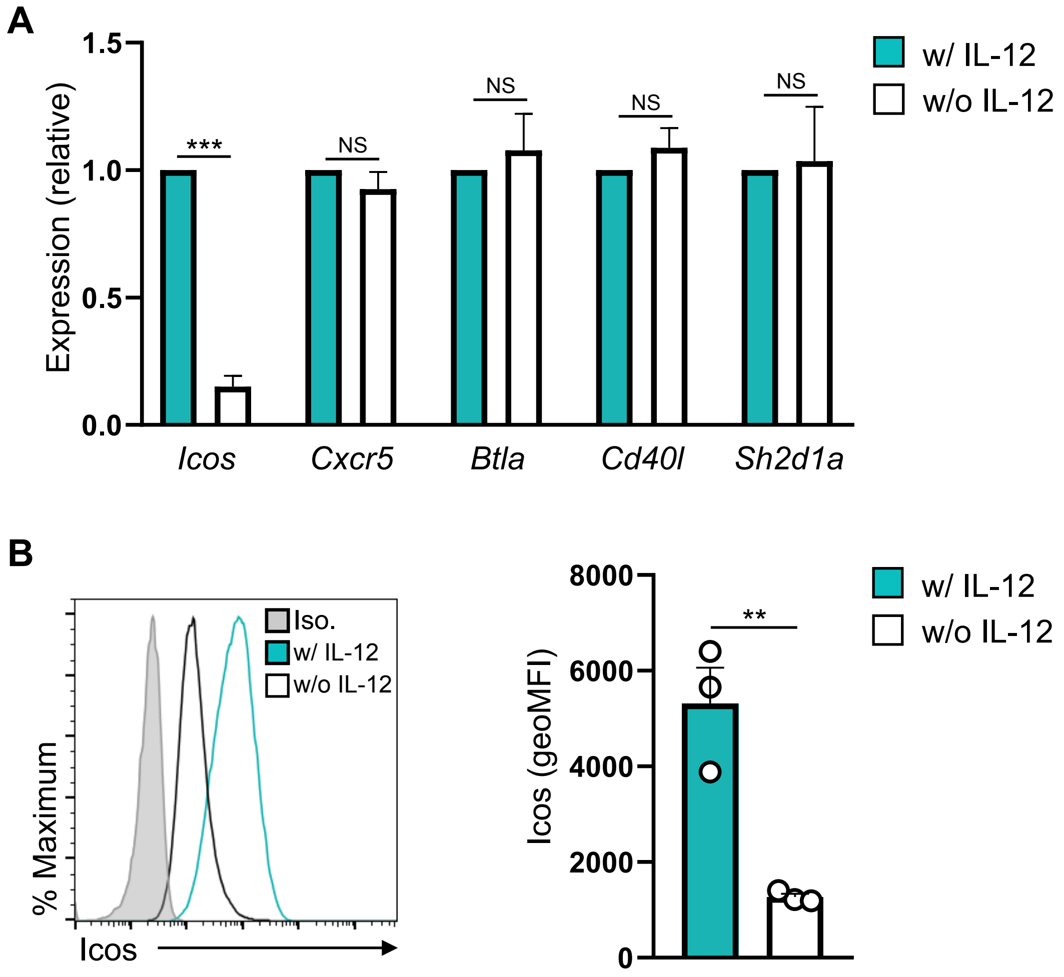

Supplementary Figure 3. Icos expression in T_{FH1}-like cells is dependent on IL-12 signaling. (A) qRT-PCR analysis of indicated genes in T_{FH1}-like cells cultured in the presence and absence of IL-12. Samples were normalized to *Rps18* and are represented relative to the T_{FH1}-like sample cultured with IL-12 (mean of n = 3 ± s.e.m.). **(B)** Flow cytometry analysis of Icos expression on T_{FH1}-like cells cultured with or without IL-12. Shown are representative data from three independent experiments. Mean fluorescence intensity (MFI) is also shown (mean of n = 3 ± s.e.m.). **P < 0.01, ***P < 0.001; unpaired Student's *t*-test.

Supplementary Figure 4

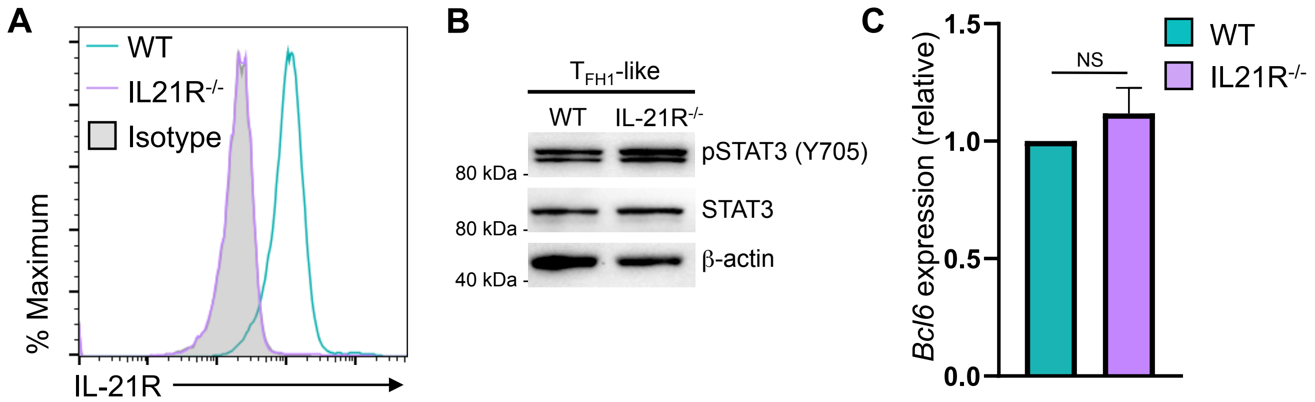

Supplementary Figure 4. STAT3 activation and *Bcl6* expression are independent of autocrine signals from IL-21 in T_{FH1}-like cells. (A) Flow cytometry analysis of IL-21R cell surface expression on wildtype (WT) and IL-21R$^{-/-}$ T_{FH1}-like cells (to confirm IL-21R deletion). Shown are representative data from three independent experiments. **(B)** Immunoblot analysis of STAT3 activation (pSTAT3 Y705) in WT and IL-21R$^{-/-}$ T_{FH1}-like cells. STAT3 and β-actin are shown as controls for total STAT3 and equal protein loading, respectively. Image shown is representative of three independent experiments. **(C)** qRT-PCR analysis of *Bcl6* transcript expression in WT and IL-21R$^{-/-}$ T_{FH1}-like cells. Data were normalized to *Rps18* and are presented relative to the WT sample (mean of n = 3 ± s.e.m.).

Supplementary Figure 5

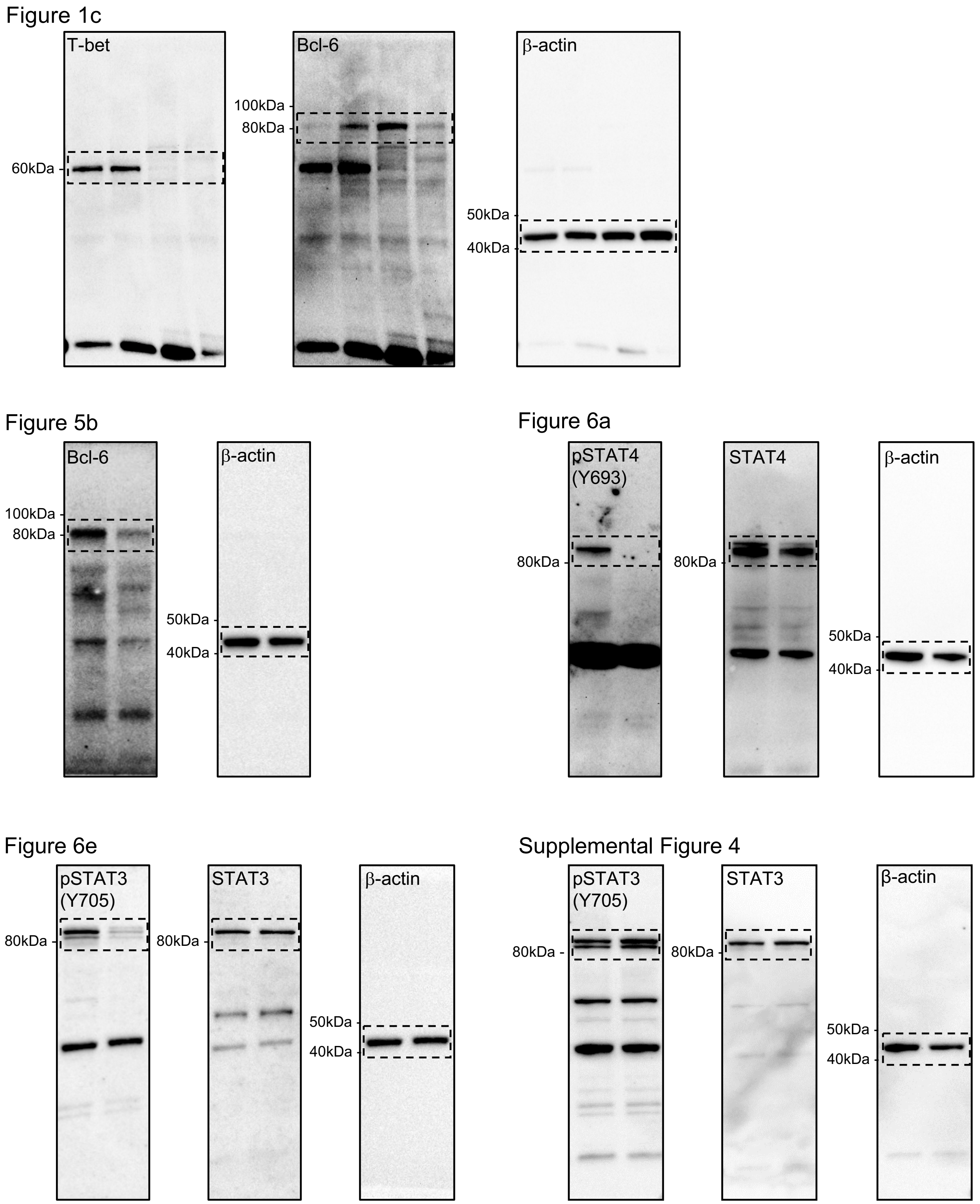

Supplementary Figure 5. Original and uncropped scans of immunoblots.
